# P10. Concomitant gemcitabine therapy negatively affects DC vaccine-induced CD8+ T cell and B cell responses but improves clinical efficacy in a murine pancreatic carcinoma model

**DOI:** 10.1186/2051-1426-2-S2-P1

**Published:** 2014-03-12

**Authors:** C Bauer, A Sterzik, F Bauernfeind, P Duewell, C Conrad, R Kiefl, S Endres, A Eigler, M Schnurr, M Dauer

**Affiliations:** 1 Medizinische Klinik und Poliklinik IV LMU Munich, Section of Gastroenterology, Munich, Germany; 2 Division of Clinical Pharmacology LMU Munich, Munich, Germany; 3 Massachusetts General Hospital Harvard University, Department of Surgery, Boston, MA, USA; 4 Klinikum Dritter Orden, Department of Clinical Medicine I, Munich, Germany; 5 Kliniken St. Elisabeth, Department of Medicine II, Neuburg/Donau, Germany

## Background

Multiple studies have shown that dendritic cell (DC)-based vaccines can induce antitumor immunity. Previously, we reported that gemcitabine enhances the efficacy of DC vaccination in a mouse model of pancreatic carcinoma. The present study aimed at investigating the influence of gemcitabine on vaccine-induced anti-tumoral immune responses in a syngeneic pancreatic cancer model.

## Material and methods

Subcutaneous or orthotopic pancreatic tumours were induced in C57BL/6 mice using Panc02 cells expressing the model antigen OVA (PancOVA). Bone marrow-derived DC were loaded with soluble OVA protein (OVA-DC). Animals received gemcitabine twice weekly. OVA-specific CD8^+^ T cells and antibody titers were monitored by FACS analysis and ELISA, respectively.

## Results

Gemcitabine enhanced clinical efficacy of the OVA-DC vaccine. Interestingly, gemcitabine significantly suppressed the vaccine-induced frequency of antigen-specific CD8^+^ T cells and antibody titers. DC migration to draining lymph nodes and antigen cross-presentation were unaffected. Despite reduced numbers of tumour-reactive T cells in peripheral blood, *in vivo* cytotoxicity assays revealed that CTL-mediated killing was preserved. *In vitro* assays revealed sensitization of tumour cells to CTL-mediated lysis by gemcitabine. In addition, gemcitabine facilitated recruitment of CD8^+^ T cells into tumors in DC-vaccinated mice. T and B cell suppression by gemcitabine could be avoided by starting chemotherapy after two cycles of DC vaccination.

## Conclusions

Gemcitabine enhances therapeutic efficacy of DC vaccination despite its negative influence on vaccine-induced T cell proliferation. Quantitative analysis of tumour-reactive T cells in peripheral blood may thus not predict vaccination success in the setting of concomitant chemotherapy.

